# Conformational Adaptation of Asian Macaque TRIMCyp Directs Lineage Specific Antiviral Activity

**DOI:** 10.1371/journal.ppat.1001062

**Published:** 2010-08-19

**Authors:** Laura M. J. Ylinen, Amanda J. Price, Jane Rasaiyaah, Stéphane Hué, Nicola J. Rose, Flavia Marzetta, Leo C. James, Greg J. Towers

**Affiliations:** 1 University College London Medical Research Council Centre for Medical Molecular Virology, London, United Kingdom; 2 Division of Infection and Immunity, University College London, London, United Kingdom; 3 Medical Research Council Laboratory of Molecular Biology, Protein and Nucleic Acid Chemistry Division, Cambridge, United Kingdom; 4 The National Institute for Biological Standards and Control, A Centre of the Health Protection Agency, Potters Bar, Hertfordshire, United Kingdom; University of Geneva, Switzerland

## Abstract

TRIMCyps are anti-retroviral proteins that have arisen independently in New World and Old World primates. All TRIMCyps comprise a CypA domain fused to the tripartite domains of TRIM5α but they have distinct lentiviral specificities, conferring HIV-1 restriction in New World owl monkeys and HIV-2 restriction in Old World rhesus macaques. Here we provide evidence that Asian macaque TRIMCyps have acquired changes that switch restriction specificity between different lentiviral lineages, resulting in species-specific alleles that target different viruses. Structural, thermodynamic and viral restriction analysis suggests that a single mutation in the Cyp domain, R69H, occurred early in macaque TRIMCyp evolution, expanding restriction specificity to the lentiviral lineages found in African green monkeys, sooty mangabeys and chimpanzees. Subsequent mutations have enhanced restriction to particular viruses but at the cost of broad specificity. We reveal how specificity is altered by a scaffold mutation, E143K, that modifies surface electrostatics and propagates conformational changes into the active site. Our results suggest that lentiviruses may have been important pathogens in Asian macaques despite the fact that there are no reported lentiviral infections in current macaque populations.

## Author Summary

Retroviruses have constantly been infecting mammals throughout their evolution, causing them to evolve defensive mechanisms to protect themselves. One of these mechanisms utilises intracellular antiviral molecules referred to as restriction factors. Restriction factor sequences have changed through primate evolution, suggesting an ongoing battle between retroviruses and their hosts as described by the Red Queen hypothesis. TRIM5 is an important restriction factor able to protect some monkeys, but not humans, from HIV infection. Certain monkeys have modified their TRIM5 genes by swapping the virus binding B30.2 domain with a cyclophilin A domain inserted into the TRIM5 locus by retrotransposition. This leads to expression of a TRIMCyp protein with antiviral activity against viruses, such as HIV-1, that recruit cyclophilins. It appears that cyclophilin makes a particularly flexible virus-binding domain able to restrict divergent lentiviruses from primates as well as cats. Here we characterise the molecular details of Cyclophilin-Capsid interactions focusing on TRIMCyp proteins from Macaca Fascicularis. Using a structure/function approach we can show the molecular details of how adaptive changes in the TRIMCyp sequence switch specificity between members of different primate lentiviral lineages. Mapping these changes onto the macaque phylogeny reveals a history of TRIMCyp evolution that directs restriction to a variety of diverse lentiviruses.

## Introduction

Mammals have evolved antiviral proteins called restriction factors, which contribute to their protection from pathogenic viral infections. Expression of restriction factors is invariably enhanced by the innate immune cytokines of the type one interferon family suggesting that restriction factors are an integral part of the innate immune system [1]. Pathogenic viral infections are thought to be a significant source of selective pressure on restriction factor evolution. Evidence for positive selection is found in the sequences of the intracellular antiviral restriction factors APOBEC3G, TRIM5α and tetherin and the positively selected amino acids have been shown to influence antiviral specificity [2]-[5]. Positions under positive selection tend to be in patches on the protein that directly contact the pathogen. Mutation of these residues alters which viruses are restricted. Variability and evidence for positive selection in regions of contact between host and pathogen illustrate the evolutionary conflict during which both constantly evolve under pressure from the other, with each alternately gaining the advantage. This ongoing arms race is described by the Red Queen hypothesis [6], [7] which has also been elegantly demonstrated by the study of bacteria/phage coevolution [8].

The restriction factor TRIM5α contains an N terminal tripartite motif comprising RING, Bbox2 and coiled coil domains and a C terminal PRYSPRY or B30.2 sequence that constitutes the virus-binding domain [9]–[13]. TRIM5α exhibits potent species-specific antiviral activity against retroviruses. This activity is mediated, in part, by recruiting proteasomes to incoming retroviral capsids leading to their premature uncoating and destruction [13]–[16]. This is observed as a potent and early block to viral DNA synthesis by reverse transcription. TRIM5α dimers are thought to recruit to the retrovirus via interactions between their B30.2 domain and retroviral capsid molecules [17]. Patches of amino acids with evidence for positive selection are found in the B30.2 domain in exposed loops on the very end of the molecule [3], [18]. The differences between species variants of TRIM5α in this region dictate antiviral specificity by determining which capsids can be recruited. Similarly, sequence differences between the viral capsids from various retroviruses, particularly in the exposed loop region referred to as the cyclophilin binding loop, influence sensitivity of the virus to restriction by TRIM5α, presumably by influencing the ability of TRIM5α to bind [10]–[12], [19].

**Figure 1:**
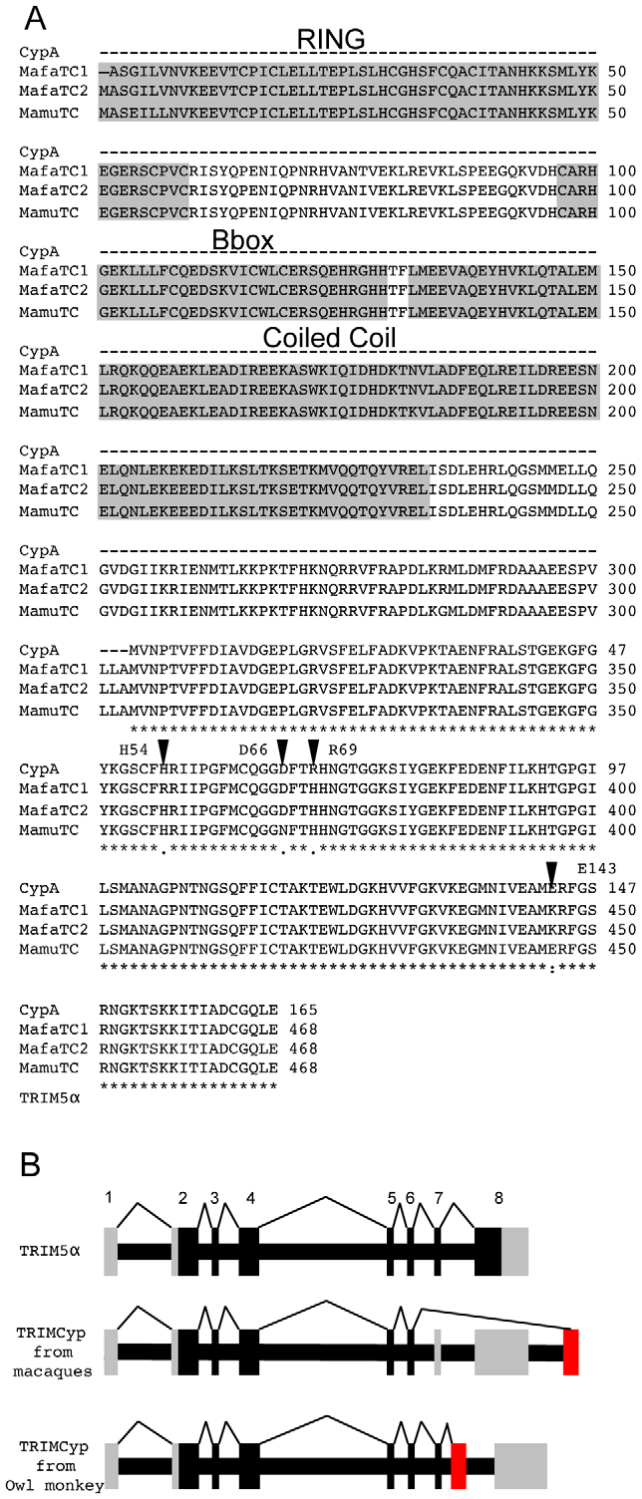
Sequence comparison of CypA and TRIMCyps from *Macaca mulatta* and *Macaca fascicularis* reveal differences in the Cyp domain of species variants of TRIMCyps. (**A**) The protein sequence of *Macaca mulatta* Cyclophilin A (Mamu CypA) AF023861 is shown aligned to TRIMCyp from *Macaca mulatta* EU157763 and *Macaca fascicularis* (Mafa) EU371638. The protein sequence of human CypA is identical. MafaTRIMCyp1 was described by Brennan and colleagues [24] and Mafa TRIMCyp2 FJ609415 is described herein. Similarity is shown by: asterisk, identical residue; colon, conserved substitution; period, semi-conserved substitution; gap, no conservation; dash, missing residue. The core domains of the RING, B-box and coiled coil regions are shown. Arrows denote positions considered in this study. CypA positions 54, 66, 69 and 143 are homologous to TRIMCyp positions 357, 369, 372 and 446 respectively. (**B**) A cartoon showing TRIMCyp splicing. TRIM5α is encoded by an 8 exon gene with 7 coding exons. In certain Old World macaque species a TRIMCyp encoding gene is found in which exons 7 and 8 are replaced by a cyclophilin A cDNA placed in the TRIM5 locus by retrotransposition [22]-[26]. The TRIMCyp found in New World owl monkeys in also shown [20], [21]. In this case the CypA sequence is in intron 7 causing replacement of exon 8 with CypA. The different positions of the CypA encoding exons in owl monkeys and macaques indicates independent evolution of Old World and New World TRIMCyp genes. Coding exons are shown in black, non coding in grey and the retrotransposed CypA cDNA is shown in red.

TRIM5-CypA chimeric proteins, referred to as TRIMCyps, have evolved through modification of the TRIM5 genetic locus on two independent occasions during primate evolution. [20]–[26]. Retrotransposition events, indicated by typical target site duplications, have inserted complete CypA cDNA sequences into the TRIM5 loci in different positions in New World owl monkeys and Old World macaques. These modifications have caused replacement of the virus binding B30.2 domain with a cyclophilin A (CypA) cDNA (Fig. 1A) CypA is a ubiquitous and highly conserved protein, whose sequence is identical in humans and macaques [27]. However, the CypA sequences in owl monkey and macaque TRIMCyps are not the same. Furthermore, like the parental CypA domain, owl monkey TRIMCyp targets HIV-1 M and O-group viruses but not HIV-2, whereas rhesus macaque TRIMCyp targets HIV-2 and HIV-1 O group but not HIV-1 M group virus. HIV-1 and HIV-2 are derived from zoonoses of different lineages of primate lentivirus, HIV-1 from chimpanzees and HIV-2 from sooty mangabeys [28], [29]. HIV-1 O and M group viruses are also distinct viruses, derived from different zoonotic transfers from chimpanzees and with radically different frequencies in the human population [29]. Recently, we showed that the change in lentiviral specificity in rhesus macaque TRIMCyp is mediated by two mutations at positions 369 and 372 (equivalent to positions 66 and 69 of the Cyp domain) that alter the conformation of the active site [30]. From hereon we use the CypA numbering for example TRIMCyp residue 369 is referred to as Cyp 66. Importantly, previous work showed that TRIMCyp encoding a Cyp domain changed at a single amino acid from the natural cyclophilin A sequence (Cyp R69H) restricts a broader range of viruses, including HIV-1 M and O groups and HIV-2 [30]. Since a broad anti-viral specificity would confer a selective advantage, we hypothesised that a TRIMCyp encoding this single change may be present as a natural allele. Investigation of naturally occurring TRIMCyp alleles in Asian Macaques reveals that an ancestral TRIMCyp bearing this broad-specificity mutation (Cyp R69H) has been specialised towards different lentiviruses in different species, giving rise to distinct specificities in present day macaques.

## Results

A TRIMCyp encoding the mutation R69H but not D66N in its Cyp domain has previously been identified in *Macaca fascicularis* [24]. To characterise *Macaca fascicularis* TRIMCyp (Mafa TRIMCyp) we RT PCR cloned a TRIMCyp cDNA from RNA purified from peripheral blood lymphocytes from an Indonesian Cynomolgus macaque. The protein sequence of the Mafa TRIMCyp cDNA that we amplified differed from that described by Brennan and colleagues at 4 positions I77T, E209K, D247E, and H357R (CypH54R). We refer to Brennan's allele as Mafa TRIMCyp1 and the allele we cloned as Mafa TRIMCyp2. The protein sequences of the two Mafa TRIMCyps are shown aligned to the rhesus macaque (*Macaca mulatta*) (Mamu) TRIMCyp and CypA protein sequences (Fig. 1).

**Figure 2:**
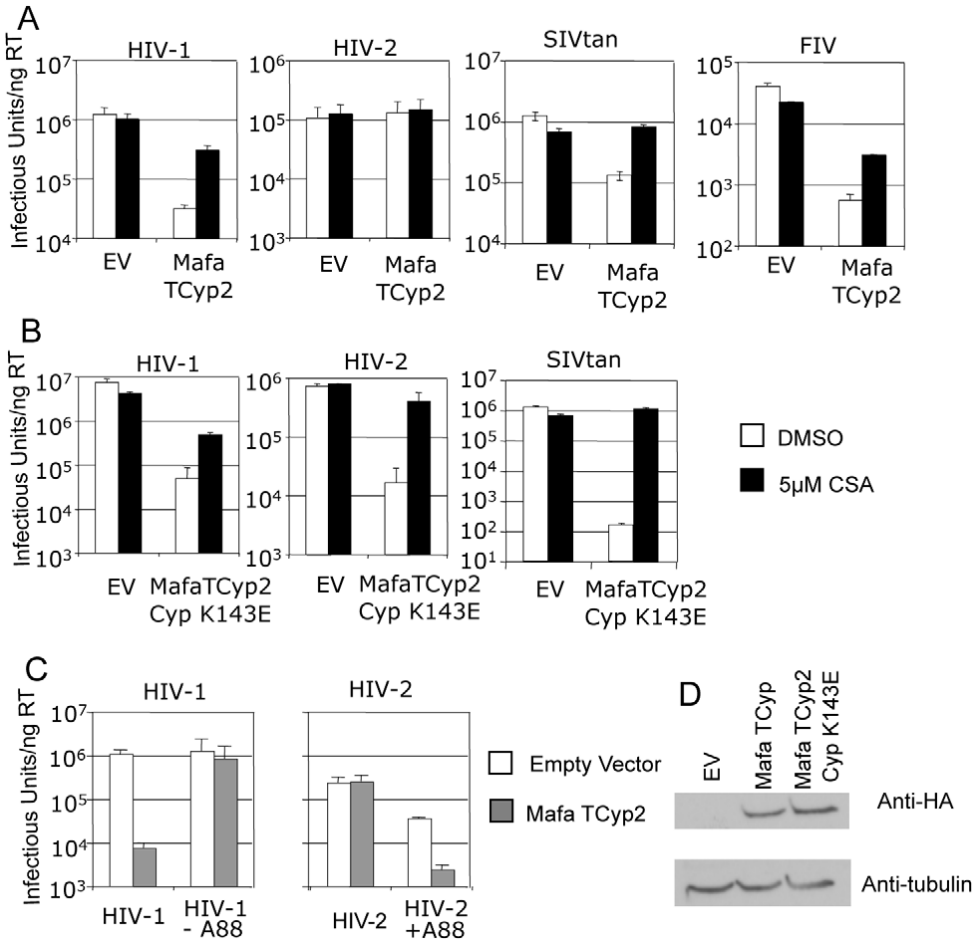
Restriction specificity of TRIMCyps against HIV-1, HIV-2 and FIV. (**A**) Feline CRFK cells expressing empty vector (EV) or Mafa TRIMCyp2 (TCyp2) were infected with GFP encoding VSV-G pseudotyped vectors derived from HIV-1, HIV-2, FIV or SIVtan as shown. White bars represent titres in the presence of DMSO vehicle and black bars represent titres determined in the presence of 5 µM cyclosporine (Cs). (**B**) As above except cells expressed the Mafa TRIMCyp2 Cyp mutant K143E (K446E in TRIMCyp). (**C**) CRFK cells expressing empty vector (white bars) or Mafa TRIMCyp2 (grey bars) were infected with wild type HIV-1 or HIV-2 vectors or HIV-1 mutant missing CA residue A88 (HIV-1 –A88) or HIV-2 mutant encoding an extra alanine at position 88 (HIV-2 +A88). (**D**) Mafa TRIMCyp expression levels in the CRFK cells were shown to be equal by western blot detecting the N terminal haemagglutinin epitope tag. The membrane was stripped and reprobed detecting tubulin to ensure equal loading. Data are representative of two independent experiments performed with independently prepared virus preps.

In order to examine the antiviral specificity of Mafa TRIMCyp2 we cloned it into a murine leukaemia virus (MLV) based expression vector and expressed it in feline CRFK cells as described [22]. These cells naturally encode a truncated TRIM5 and are thus highly permissive to VSV-G pseudotyped, GFP encoding, lentiviral vectors and exhibit no detectable post-entry restriction activity to retroviral vectors [31], [32]. We infected the Mafa TRIMCyp2 expressing CRFK cells with lentiviral vectors derived from HIV-1, HIV-2, Simian Immunodeficiency Virus from Tantalus monkeys (SIVtan) and Feline Immunodeficiency Virus (FIV) as described [33], [34]. CRFK expressing empty vector were infected as a control and infected cells were enumerated 48 hours later by flow cytometry (Fig. 2A). Mafa TRIMCyp2 restricted HIV-1 and SIVtan but not HIV-2 infectivity. Mafa TRIMCyp2 also restricted FIV infectivity. Furthermore, HIV-1, SIVtan and FIV infectivity were rescued by adding the CypA inhibitor cyclosporine (Cs) at 5 µM, as has been described [22], [23].

We were surprised that Mafa TRIMCyp2 restricted HIV-1 and not HIV-2 because a previous study has shown that Mamu TRIMCyp protein encoding identical residues at Cyp domain positions 66 (aspartate) and 69 (histidine) can restrict both viruses [30]. Comparison of the Mafa and Mamu sequences (Fig. 1) indicates that the Mafa TRIMCyp proteins differ from the Mamu TRIMCyp sequence in the CypA domain at position Cyp 143, encoding a lysine rather than a glutamate. We therefore mutated the Mafa TRIMCyp2 to resemble the Mamu sequence at this position (Cyp K143E) and expressed it in CRFK cells as before. Cells expressing the MafaTRIMCyp2 Cyp K143E were able to restrict both HIV-1 and HIV-2 by around 2 orders of magnitude (Fig. 2B). This TRIMCyp mutant was also much more potent against SIVtan infectivity. Treatment with 5 µM Cs partially rescued HIV-1 and SIVtan infectivity and completely rescued HIV-2 infectivity (Fig. 2B). We hypothesised that the Cyp E143K (E446K) mutation specifically prevents binding of the HIV-2 capsid, whilst HIV-1 binding is unaffected. To test this, we measured interaction between the HIV-1 and HIV-2 N-terminal capsid domains and the CypA domains from the Mafa TRIMCyp2 allele and K446E (Cyp K143E) mutant by isothermal titration calorimetry (ITC). Whilst the K143E mutant bound both HIV-1 and HIV-2, Mafa TRIMCyp2 was only able to bind HIV-1 (Table 1). These data demonstrate that the Cyp E143K change in Mafa TRIMCyp2 switches specificity from being able to bind and restrict HIV-1 and HIV-2 to being able to bind and restrict only HIV-1. Cyp E143K also influences antiviral specificity against the African green monkey lineage virus SIVtan, restricting it much more weakly than its ancestral TRIMCyp protein. This change therefore influences antiviral activity against members of three lineages of extant primate lentiviruses.

**Table 1:** Affinities of cyclophilin domain binding to N terminal domain capsids, as measured by isothermal titration calorimetry, are shown.

	HIV-1	HIV-2	HIV-1-A88	HIV-2+A88	FIV
**Mafa TCyp2**	12 µM	no binding	no binding	207 µM	27 µM
**Mafa TCyp2 K143E**	12 µM	13 µM	no binding	9 µM	15 µM

In a previous study comparison of the crystal structure of the N terminal capsid domain of HIV-2 with HIV-1 indicated that the HIV-2 CypA binding loop is shorter by a single alanine residue at the position equivalent to HIV-1 CA88 [30]. This underlies a significant conformational difference between the CypA binding loops of HIV-1 and HIV-2 capsids and dictates binding to TRIMCyp variants, which in turn correlates with restriction sensitivity. We therefore tested whether the alanine at CA88 also dictated sensitivity to Mafa TRIMCyp. To do this we used a previously described HIV-1 mutant in which the alanine at CA88 had been removed (HIV-1 CA-A88), and an HIV-2 mutant in which the missing alanine has been inserted (HIV-2 CA+A88). GFP encoding HIV-1 and HIV-2 mutant vectors were prepared and used to infect CRFK cells expressing Mafa TRIMCyp2 and cells bearing empty vector as a control (Fig. 2C). In fact, whilst HIV-1 was sensitive to Mafa TRIMCyp2 the mutant with a shorter CypA binding loop was unrestricted. Furthermore, Mafa TRIMCyp2 restricted the HIV-2 mutant with an extra alanine, whereas the wild type virus remained unrestricted. The HIV-1 mutant was as infectious as the wild type virus whereas the HIV-2 mutant with an extra alanine was around an order of magnitude less infectious than wild type on feline cells bearing empty vector. However, the switch in sensitivity to TRIMCyp on adding or removing the extra alanine residue in these viruses is clear. Furthermore, corresponding binding experiments by ITC support the restriction data; the loss or gain of restriction sensitivity upon addition or removal of A88 directly correlates with loss or gain of binding (Table 1). Sensitivity to Mafa TRIMCyp is therefore dictated by the sequence of the CypA binding loop with the alanine at CA88 being an important determinant.

**Figure 3:**
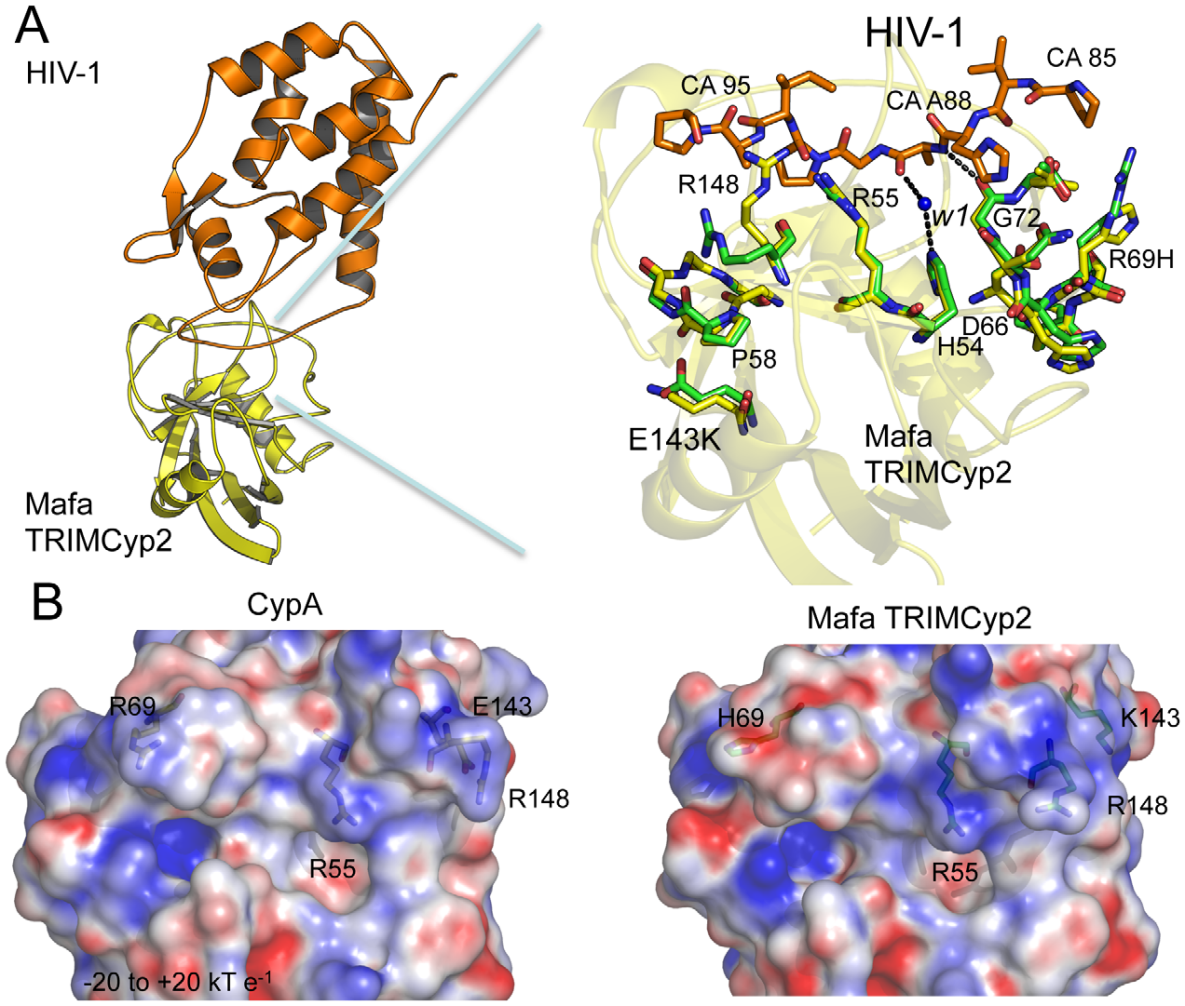
Crystal structure of the Mafa Cyp:HIV-1 N-terminal capsid domain complex. (**A**) A secondary structure representation of the complex is shown (top left) together with a detailed view of the active site (top right). The detailed view shows a superposition with CypA side chains from the HIV-1:CypA complex (1AK4) coloured green and the Cyp side chains from Mafa TRIMCyp2 coloured yellow. Interacting residues from the HIV-1 CypA-binding loop are coloured orange. The secondary structure of Mafa Cyp is coloured yellow in the background. Cyp mutation E143K propagates structural changes within the active site around P58 and R148. Position 88 in the HIV-1 capsid makes several key interactions indicated as dashed lines, including via a water molecule (W1) conserved in both wild-type and E143K structures. Mutation R69H does not alter the backbone conformation of the loop. Mutation E143K leads to some alteration of backbone (P58) and surrounding side-chains (R148). Position 88 in the HIV-1 capsid makes several key interactions indicated as dashed lines, including with a water mediated interaction via a water molecule conserved in both wild-type and E143K structures. (**B**) Comparison of electrostatic surface potential of CypA and Cyp domains from Rhesus and Mafa TRIMCyps. The effect of mutations R69H and E143K on the electrostatic surface potential of the Cyp active site is shown. Charges were calculated and mapped onto the molecular surface using APBS and scaled from -20 to +20 kT e^−1^. Blue represents a positive charge and red negative.

In order to understand the structural basis for these restriction specificities we solved the crystal structure of the Mafa TRIMCyp2 Cyp domain in complex with the HIV-1 capsid N terminal domain (NTD) (Table S1). As stated above, there are two amino acid differences between the Cyp domain of Mafa TRIMCyp and the genomic CypA sequence, R69H and E143K. Superposition of the Mafa:HIV-1 complex with the previously solved wild-type CypA:HIV-1 structure (1AK4) [35] reveals that the two Cyp mutations (R69H and E143K) do not provoke large structural rearrangements (Fig. 3A). This is in contrast to the two mutations in Cyp from Mamu TRIMCyp (D66N and R69H) that have previously been shown to create a large (>16 Å) rearrangement of the active site loop around positions 66–69 (loop_66–69_) and switch specificity of binding and restriction from HIV-1 M group to HIV-2 [30]. Mafa TRIMCyp thus maintains binding to HIV-1 despite the R69H mutation because it conserves the closed loop_66–69_ conformation observed in wild type CypA. The closed-loop conformation allows interactions with capsid position CA88, enabling several hydrogen bond interactions to be made, including between the CA88 peptide nitrogen and the peptide oxygen from G72 in Cyp and a water-mediated interaction between the peptide oxygen of CA88 and Cyp residue H54. The loss of these interactions resulting from removal of A88 from HIV-1 explains why this mutant virus neither binds nor is restricted by Mafa TRIMCyp2 (Fig. 3A).

The E143K mutation is located in a helix outside of the active site at the opposite end of the Cyp domain to R69H. How does such a distant mutation impact on viral specificity? The Mafa complexed structure shows that the change from a negatively charged glutamate to a positively charged lysine at 143 causes a knock on effect through the molecule which impacts on the stereochemistry of the active site. Mutation to lysine at position 143 causes changes to the backbone of a neighbouring active-site loop around P58 by attracting the peptide oxygen and flipping it from its orientation in the wild-type CypA structure (Fig. 3A). This propagates changes to other Cyp active-site residues, including R148 that is coordinated by the P58 peptide oxygen in the wild-type structure (Fig. 3A). The altered position of P58 releases the side chain of R148, allowing it to interact with capsid residues downstream of residue A88. The E143K mutation also modifies the surface electrostatics (Fig. 3B) leading to a significant increase in the positive charge of the active site. Surface electrostatics are also shown for rhesus TRIMCyp and CypA for comparison. E143K therefore impacts on viral specificity through propagated conformational changes and long-range electrostatic effects.

**Figure 4:**
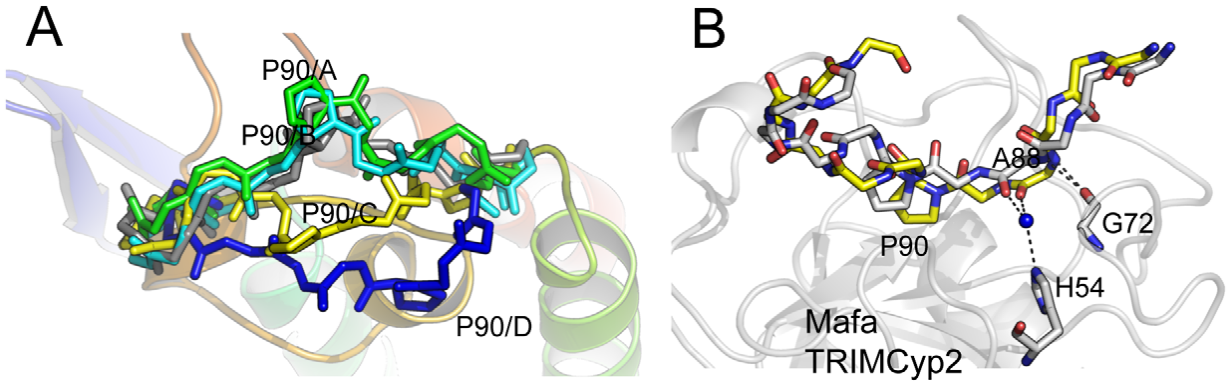
CypA-binding loop conformations in the HIV2 +A88 capsid structure. (**A**) The four copies of the capsid in the asymmetric unit have been superposed and the main chain of the CypA-binding loop shown. In gray is the structure of the HIV-2 capsid without A88 inserted. (**B**) Superposition of HIV1:CypA complex and HIV-2 +A88 capsid structures. The main-chain of the CypA-binding loop of HIV-1 is shown in gray and HIV-2 +A88 in yellow. Insertion of A88 into HIV-2 may allow hydrogen bond interactions between capsid residues H54 and G72 as seen in the HIV-1 complex.

HIV-1 binding is unaffected by the charge alterations introduced by R69H and E143K (Fig. 3B), whereas binding to HIV-2 is conferred by a reduction in positive charge (R69H) and abrogated by an increase in positive charge (E143K). HIV-2 can be made insensitive to these charge changes by introducing A88 into the capsid, resulting in susceptibility to Mafa TRIMCyp2. This suggests that the two viruses normally utilise different binding mechanisms but that insertion of A88 confers an HIV-1 like binding mode. To test this we solved the structure of HIV-2 with an alanine inserted at position 88 (Table S1). The introduction of A88 increases the structural dynamics of the HIV-2 CypA-binding loop and allows it to adopt alternative conformations (Fig. 4A). Importantly, this allows the main-chain nitrogen of the introduced A88 to adopt an orientation that in HIV-1 permits hydrogen bond interaction with cyclophilin residues G72 and H54 (Fig. 4B). These hydrogen bond interactions are not affected by the E143K mutation, explaining why HIV-2 with an inserted A88 is no longer affected by the charge change. It is unlikely that these interactions are normally possible in HIV-2, since it has a deletion at this position and the preceding proline lacks a hydrogen on its amide group. Position CA88 is therefore a critical determinant of sensitivity even to mutations that are over 20 Å away as in the E143K Mafa TRIMCyp2 allele.

**Figure 5:**
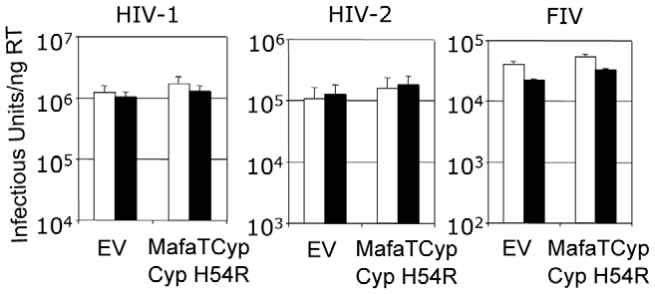
Mutation H54R in the Cyp domain abrogates Mafa TRIMCyp2's antiviral activity against HIV-1 and FIV. Feline CRFK cells expressing empty vector (EV) or Mafa TRIMCyp2 bearing Cyp mutation H54R were infected with GFP encoding VSV-G pseudotyped vectors derived from HIV-1, HIV-2 or FIV as shown. White bars represent titres in the presence of DMSO vehicle and black bars represent titres determined in the presence of 5 µM cyclosporine (Cs). The Cyp domain mutation H54R found in Mafa TRIMCyp1 explains its described lack of activity against HIV-1 [24]. Data are representative of two independent experiments performed with independently prepared virus preps.

In contrast to Mafa TRIMCyp2's ability to restrict HIV-1 the Mafa TRIMCyp1 protein identified by Brennan and colleagues was shown to not restrict HIV-1 [24]. Mafa TRIMCyp 1 and 2 differ at four positions including Cyp position 54. We therefore tested whether this difference is responsible for Mafa TRIMCyp1's lack of anti-HIV-1 activity. We mutated Cyp H54 to arginine in Mafa TRIMCyp2 and expressed it in CRFK cells. Infection of the modified cells with HIV-1, HIV-2 and FIV derived GFP encoding vectors indicated that Cyp H54R ablates Mafa TRIMCyp2's ability to restrict both HIV-1 and FIV, explaining the different restriction activities (Fig. 5). HIV-2 is also unrestricted by Mafa TRIMCyp2 Cyp H54R. Consideration of the Cyp-CA complex structure (Fig. 3A) explains this observation. The peptide oxygen of CA88 makes a water-mediated interaction with cyclophilin residue H54 (H357 in TRIMCyp). The importance of this interaction is indicated by the fact that the water molecule is conserved in both the wild-type (1AK4) [35] and E143K structures (Fig. 3A).

**Figure 6:**
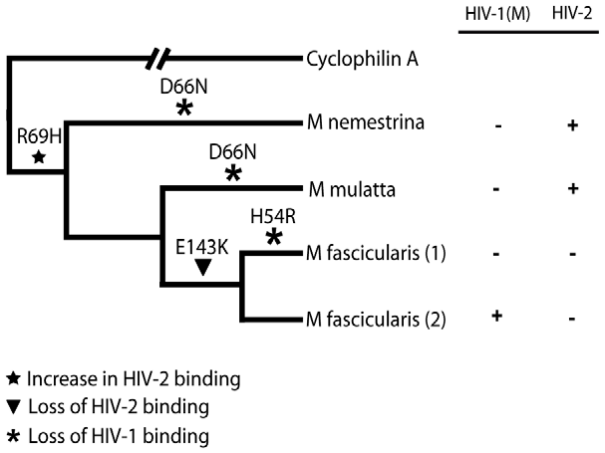
Mapping the differences in the TRIMCyp cyclophilin sequences onto the macaque phylogeny suggests convergent evolution of D66N and switching of antiviral specificity between lentiviral lineages throughout Asian macaque evolution. The macaque phylogeny is shown with the changes marked on the branches. The table indicates restriction specificity with crosses indicating strong restriction. Restriction broadly correlates with Cyp-CA binding (Fig. 2, Table 1) [30]. CypA binds HIV-1 strongly and HIV-2 weakly [30].

In order to consider the differences in Asian Macaque TRIMCyp sequence in the context of Asian macaque evolution, we mapped the changes onto the recently described macaque phylogeny [36] (Fig. 6). We assume that the CypA sequence retrotransposed into the macaque TRIM5 locus was wild type. TRIMCyp with a wild type CypA sequence has been shown to restrict HIV-1 but HIV-2 only weakly [23], [30]. Intriguingly, the phylogeny suggests that the R69H mutation arose early in a macaque common ancestor, conferring broad antiviral specificity including against both HIV-1 and HIV-2. Following speciation events giving rise to present day macaque species, subsequent TRIMCyp mutations were either selected or underwent fixation. D66N mutation may have arisen independently in two different macaque species, *Macaca mulatta* and *Macaca nemestrina*. This is suggested by the fact that the D66N change is not found in *Macaca fascicularis* TRIMCyps (Fig. 1). D66N switches antiviral activity from restriction of both HIV-1 and HIV-2 to restriction of HIV-2 only (Fig. 2). In contrast, the *Macaca fascicularis* TRIMCyp sequence has acquired an alternate change, E143K, which switches antiviral specificity from HIV-1 and HIV-2 to HIV-1 alone, the opposite direction to *nemestrina/mulatta* (Figs. 2–3). The allelic diversity in present day macaque species represents functional TRIMCyps of defined lineage-specificity, strongly suggesting selection pressure from different lentiviruses.

## Discussion

Here we have considered the restriction of HIV-1, HIV-2 and SIVtan as well as the feline lentivirus FIV by TRIMCyp proteins from Cynomolgus macaques (*Macaca fascicularis*). Analysis of TRIMCyp antiviral specificity has revealed that a surprising diversity of antiviral activity can be achieved using a cyclophilin A-like domain to target lentiviral capsids. The antiviral activity of TRIMCyps can be very specific, distinguishing between HIV-1 and HIV-2, which are members of different lentiviral lineages, as well as between HIV-1 M and O group viruses, which are derived from independent zoonoses of SIVcpz from chimpanzees to humans [23], [30]. TRIMCyps can thus distinguish between closely related lentiviruses for example the HIV-1s, whilst also being able to restrict distantly related lentiviruses, for example FIV. By studying the details of the structural changes that underlie the switching of antiviral specificity we can gain a better appreciation of the flexibility of CypA-lentiviral interactions. The Cyp E143K change found in the TRIMCyp from Cynomolgus macaques (*Macaca fascicularis*) is surprisingly distant from the region of contact between Cyp and the lentiviral capsids. Nonetheless, by propagating structural changes across the molecule this change can switch a Cyp capable of binding and restricting HIV-1 and HIV-2 to one that only restricts HIV-1. The selection of a mutation that affects specificity through propagated changes is reminiscent of the antibody immune response. Affinity maturation has been shown to utilise distant indirect mutations to alter the specificity and affinity of more generalized primary antibodies [37]–[39].

Remarkably, the potency of single amino acid changes in Cyp is mirrored by the effect of single amino acid changes in the lentiviral capsids. Altering HIV-1 or HIV-2 by a single amino acid can switch either virus cleanly between exquisite sensitivity and complete insensitivity to restriction by Mafa TRIMCyp2 (Fig. 2C). These observations reinforce the notion that there is a fine balance between restriction and replication. We imagine that the host, which evolves relatively slowly, can compete successfully with rapidly evolving lentiviruses because it relies on a large number of innate and adaptive pathways to combat viral infection. The host can also make complex adaptive changes, illustrated for example, by the exchange of the B30.2 viral binding domain for CypA placed in the TRIM5 locus by retrotransposition.

There are three possible explanations for the present day allelic distribution of TRIMCyp in macaque species. First, modern macaque species may have originated from a common ancestor that was multi-allelic. Incomplete lineage sorting during speciation may then have given rise to the limited allelic diversity in each species. A second possibility is that following speciation, each species may have been placed under selection by a different virus, causing fixation of different alleles with different viral specificities. Finally, the macaque common ancestor may have had limited allelic diversity, for instance the broad specificity R69H allele, and different viruses subsequently fixed new mutations in emerging macaque species. In this last scenario, the D66N mutation that switches specificity to HIV-2, would represent convergent evolution in M.nemestrina and M.mulatta. It is likely that a combination of the above processes - selection and random fixation - underlie present day species diversity.

Irrespective of the evolutionary sequence of events, the data presented here illustrate that specific changes made in the Cyp domain of TRIMCyp during Asian monkey evolution make important, and surprisingly potent, alterations to its antiviral specificity [23], [30]. Given that the D66N, R69H and E143K TRIMCyp mutations all affect viral specificity it is tempting to speculate that the Mafa TRIMCyp1 allele with its H54R mutation in its Cyp domain, though not active against the lentiviruses tested, restricts viruses that we have yet to determine. Cyp position H54 is an active site residue that forms a water-mediated interaction with HIV-1 (Fig. 3A). A larger arginine side-chain could be expected to form a similar interaction but without an intermediate water. This makes H54R a good candidate for fixation through selective pressure from a viral infection in the distant past, as is likely the case for the other TRIMCyp mutations.

The conclusion that lentiviruses have exerted selection pressure in Asian macaques presupposes that Asian macaques have lost the selective virus, or viruses, permanently as there are currently no extant lentiviruses described in these animals. However, we cannot be certain that lentiviruses do not exist in Asian macaque populations as they have not been surveyed systematically and rare viruses may yet be found. The fossil record suggests that macaques migrated across Europe around 5.5 mya [36], whilst lentiviruses are known to have been present in European lagomorphs for at least 12 million years [40], [41], It is therefore possible that lentiviruses may have undergone interspecies transmission into macaques at around this time. In contrast, the Asian macaque-tropic SIVmac appears to be a cross-species transmission from sooty mangabeys that occurred in captivity and was probably the inadvertent result of forced passage experiments during investigation of kuru transmission [42], [43]. It is difficult to find evidence for the loss of a lentivirus from a species but it is clear that the numbers of HIV-1 O group infected individuals declined sharply between 1988 and 1998 in Cameroon, although the frequency now appears stable [44]. The number of HIV-2 infected individuals has also fallen suggesting that in time these viruses could disappear altogether from the human population [45]. The rarity of endogenous lentiviral sequences has suggested that lentiviruses are relatively young compared to related retroviruses that are commonly found as endogenous sequences but it may be that they are simply less efficient at entering the germ lines of their hosts [40], [41], [46]. Thus, our observations suggest that whilst lentiviruses may not be present in current populations of Asian macaques they may have been important pathogens in the past. Of course, other, as yet unidentified, cyclophilin binding pathogens could have contributed to the selective pressures driving TRIMCyp evolution although the switches in primate lentiviral specificity that we have described lead us to favour lentiviruses as an important selective force.

In conclusion, the structural changes mediated by Cyp residue E143K illustrate the remarkable adaptability of CypA as a viral binding domain, providing the necessary flexibility to respond to a rapidly changing pathogen. Seemingly innocuous scaffold mutations such as Cyp E143K can propagate structural changes within CypA, resulting in sufficient rearrangement of the active site to potently alter viral restriction specificity. The ease with which the conformation and hence specificity of CypA can be changed with even a single mutation explains why it has been selected as a targeting domain for a primate restriction factor. The characterisation of the different TRIMCyp homologues and their alleles present in the *Macaca* species *– nemestrina, mulatta* and *fascicularis –* show that each TRIMCyp mutation has a direct structural, thermodynamic and functional effect on the restriction of viruses from three lentiviral lineages. This strongly suggests that these mutations are not the effect of random drift, but instead that primate TRIMCyps have undergone positively selected changes that have impacted specifically on antiviral activity.

## Methods

### Cells and viruses and infectivity experiments

Feline CRFK cells were a gift from Yasuhiro Ikeda and were maintained in DMEM (Invitrogen) with 10% FCS (Biosera). VSV-G pseudotyped lentiviral vectors encoding GFP derived from HIV-1 [47], [48], HIV-2 [49] and FIV [50] were prepared as described [51]. SIVtan with GFP in place of Nef was a gift from Paul Bieniasz and was VSV-G pseudotyped as described [33]. Viral mutants HIV-1 CA –A88 and HIV-2 CA +88A have been described [30]. Infections were performed on 25000 cells/well in 24 well plates or 200,000 cells/well in 6 well plates. Serial dilutions of virus were used to infect cells and the percentage of green cells expressing GFP was measured 48 hours later by flow cytometry as described [52]. Data are presented as mean titres derived from two doses and errors are standard errors of the mean. Data are representative of two independent experiments. Cyclosporine (Sandoz) was diluted in DMSO and added to cells at the time of infection at 5 µM. Viral doses were measured by reverse transcriptase enzyme linked immunosorbant assay (Roche).

### Cloning Mafa TRIMCyp2

RNA was purified from peripheral blood mononuclear cells (PBMC) from an Indonesian Cynomolgus macaque (RNAeasy, Qiagen) and reverse transcribed to cDNA (Superscript, Invitrogen). Mafa TRIMCyp was then cloned by PCR using pfu turbo (Stratagene) into the murine leukaemia virus based vector EXN [53], a gift from Paul Bieniasz. Three independent clones were sequenced and the Mafa TRIMCyp2 sequence has Genbank accession number FJ609415. MLV vector was prepared and TRIMCyp expressed as described [22], [34]. Mutagenesis was performed as described [54]. EXN encodes an N terminal HA tag allowing detection of protein expression by western blot, detecting the HA tag.

### Protein expression and purification

CA^N^ domains of HIV-1 M-type (NL4.3) and HIV-2 (ROD) were expressed and purified as previously described [30]. The CypA domain from MafaTRIMCyp was expressed as an N-terminal His-tagged protein in BL21 (DE3) cells and protein purified by capture on Ni-NTA resin (Qiagen) followed by gel filtration. All mutant proteins were expressed and purified as per the wild-type proteins.

### Isothermal titration calorimetry

ITC experiments were conducted on a MicroCal ITC200. Proteins were dialysed overnight against phosphate buffer (50 mM KPO_4_ (pH 7.4), 100 mM NaCl, 1 mM DTT) and experiments carried out at 15°C, with cyclophilin (typical concentration 1.4 mM) in the syringe and capsid (typical concentration 120 µM) in the cell. Binding isotherms were fitted using the standard one-state model within the MicroCal instrument software, as previously described [30].

### Crystallography

Crystals of Mafa TRIMCyp2 CypA domain in complex with HIV-1 CA^N^ were grown at 17 °C in sitting drops. Protein solution (0.33 mM each of Mafa Cyp and HIV-1 CA^N^ in 20 mM HEPES pH 7, 50 mM NaCl, 1 mM DTT) was mixed with reservoir solution (30% PEG 6000, 1 M LiCl, 0.1 M HEPES pH 7, 0.5% ethyl acetate) in a 1:1 mix, producing 0.2 mm 5 0.12 mm 5 0.02 mm crystals within 48 h. Crystals of HIV-2 CA^N^ +A88 were grown at 17 °C in sitting drops. Protein solution (0.6 mM in 20 mM HEPES pH 7, 50 mM NaCl, 1 mM DTT) was mixed with reservoir solution (2.8 M sodium acetate trihydrate pH 7, 3% 1,4-dioxane, 1% PEG 3350) in a 1:1 mix, producing 0.08 mm 5 0.04 mm 5 0.04 mm crystals within 48 h. Data was collected on an in-house FR-E SuperBright high-brilliance rotating anode linked to an automated crystal mounting system (ACTOR; Rigaku). Data were processed and refined using programmes from the CCP4 package [55]. Molecular replacement (Phaser) was performed using structure 1AK4 [35] for the HIV1:Cyno complex and the free HIV-2 structure [30] for the HIV-2 +A88 structure. Figures were prepared using Pymol and the adaptive Poisson-Boltzmann solver (APBS) for electrostatics.

### Accession codes

Protein Data Bank: Coordinates for HIV-2 +A88 capsid mutant and Mafa TRIMCyp Cyp domain in complex the HIV-1 CA have been deposited with accession codes 2X82 and 2X83, respectively.

Mafa TRIMCyp2 sequence has Genbank accession number FJ609415.

## Supporting Information

Table S1 Data collection and refinement statistics
(0.04 MB DOC)
