# Supplementary material for: Conformational Adaptation of Asian Macaque TRIMCyp Directs Lineage Specific Antiviral Activity
Source: PLoS Pathog. 2010 Aug 19;6(8):e1001062. doi: 10.1371/journal.ppat.1001062 (PMC2924388; doi:10.1371/journal.ppat.1001062)
Supplement: Table S1 — Data collection and refinement statistics (0.04 MB DOC) [file ppat.1001062.s001.doc]

**Table S1.** **Data collection and refinement statistics**

|  | HIV-2 +A88 | HIV1:Cyno complex |
| --- | --- | --- |
| **Data collection** |  |  |
| Space group | P212121 | P1211 |
| Cell dimensions |  |  |
| *a*, *b*, *c* (Å) | 47.47 116.77 129.36 | 38.52 110.15 67.76 |
| a, b, g (°) | 90.00 90.00 90.00 | 90.00 101.59 90.00 |
| Resolution (Å) | 86 – 2.6 | 66 – 1.7 |
| *R*_sym_ | 0.098(0.512) | 0.057(0.507) |
| *I* / s*I* | 6.6(2.0) | 9.1(1.9) |
| Completeness (%) | 95.7(95.7) | 95.1(95.1) |
| Redundancy | 2.9(2.8) | 2.4(2.3) |
|  |  |  |
| **Refinement** |  |  |
| Resolution (Å) | 2.6 | 1.7 |
| No. reflections | 22894 | 60435 |
| *R*_work_ / *R*_free_ | 0.22/0.28 | 0.184/0.246 |
| No. atoms |  |  |
| Protein | 4,928 | 9,742 |
| Ligand/ion | 0 | 0 |
| Water | 58 | 1925 |
| *B*-factors |  |  |
| Protein | 34.1 | 23.9 |
| Ligand/ion | n/a | n/a |
| Water | 23.2 | 43.7 |
| R.m.s deviations |  |  |
| Bond lengths (Å) | 0.018 | 0.018 |
| Bond angles (°) | 2.2 | 2.2 |

*Values in parentheses are for highest-resolution shell.
